# Chinese expert consensus on minimally invasive interventional treatment of trigeminal neuralgia

**DOI:** 10.3389/fnmol.2022.953765

**Published:** 2022-07-28

**Authors:** Xiaochong Fan, Zhijian Fu, Ke Ma, Wei Tao, Bing Huang, Gang Guo, Dong Huang, Guangzhao Liu, Wenge Song, Tao Song, Lizu Xiao, Lingjie Xia, Yanqing Liu

**Affiliations:** ^1^Department of Pain Medicine, The First Affiliated Hospital of Zhengzhou University, Zhengzhou, China; ^2^Department of Pain Medicine, Shandong Provincial Hospital Affliated to Shandong First Medical University, Jinan, China; ^3^Department of Pain Medicine, Xinhua Hospital Affiliated to Shanghai Jiao Tong University School of Medicine, Shanghai, China; ^4^Department of Functional Neurosurgery, Shenzhen University General Hospital, Shenzhen, China; ^5^Department of Pain Medicine, The Affliated Hospital of Jiaxing University, Jiaxing, China; ^6^Department of Interventional Medicine, Lanzhou University First Hospital, Lanzhou, China; ^7^Department of Pain Medicine, The Third Xiangya Hospital of Central South University, Changsha, China; ^8^Department of Pain Medicine, The Second Hospital of Hebei Medical University, Shijiazhuang, China; ^9^Department of Pain Medicine, The First Hospital of China Medical University, Shenyang, China; ^10^Department of Pain Medicine, The Union Shenzhen Hospital of Huazhong Science and Technology University, Shenzhen, China; ^11^Department of Pain Medicine, Henan Provincial People’s Hospital, Zhengzhou, China; ^12^Department of Pain Medicine, Beijing Tiantan Hospital, Capital Medical University, Beijing, China

**Keywords:** trigeminal neuralgia, radiofrequency thermocoagulation, percutaneous microcompression, neuropathic pain, headache

## Abstract

**Background and purpose:**

Trigeminal neuralgia is a common condition that is associated with severe pain, which seriously affects the quality of life of patients. When the efficacy of drugs is not satisfactory or adverse drug reactions cannot be tolerated, minimally invasive interventional therapy has become an important treatment because of its simple operation, low risk, high repeatability and low cost. In recent years, minimally invasive interventional treatments, such as radiofrequency thermocoagulation (RF) of the trigeminal nerve and percutaneous microcompression (PMC), have been widely used in the clinic to relieve severe pain in many patients, however, some related problems remain to be addressed. The Pain Association of the Chinese Medical Association organizes and compiles the consensus of Chinese experts to standardize the development of minimally invasive interventional treatment of trigeminal neuralgia to provide a basis for its clinical promotion and application.

**Materials and methods:**

The Pain Association of the Chinese Medical Association organizes the Chinese experts to compile a consensus. With reference to the evidence-based medicine (OCEBM) system and the actual situation of the profession, the Consensus Development Committee adopts the nominal group method to adjust the recommended level.

**Results:**

Precise imaging positioning and guidance are the keys to ensuring the efficacy and safety of the procedures. RF and PMC are the most widely performed and effective treatments among minimally invasive interventional treatments for trigeminal neuralgia.

**Conclusions:**

The pain degree of trigeminal neuralgia is severe, and a variety of minimally invasive intervention methods can effectively improve symptoms. Radiofrequency and percutaneous microcompression may be the first choice for minimally invasive interventional therapy.

## Overview

Trigeminal neuralgia is a common condition that is associated with severe pain, which seriously affects the quality of life of patients. When the efficacy of drugs is not satisfactory or adverse drug reactions cannot be tolerated, minimally invasive interventional therapy has become an important treatment because of its simple operation, low risk, high repeatability and low cost. In recent years, minimally invasive interventional treatments, such as RF of the trigeminal nerve and PMC, have been widely used in the clinic to relieve severe pain in many patients, however, some related problems remain to be addressed.

In order to standardize the development of minimally invasive interventional treatment for trigeminal neuralgia and provide a basis for its clinical promotion and application, The pain Association of Chinese Medical Association organized Chinese experts to compile a consensus with reference to evidence-based medicine (OCEBM) system and the actual situation of the profession, using the nominal grouping method to adjust the recommendation level (Table 1).

**TABLE 1:** Oxford University Center for Evidence-Based Medicine (OCEBM) evidence level and recommendation level standard.

Level of evidence	Definition
1a	Systematic review of randomized controlled trials (homogeneity)
1b	Individual randomized controlled trials (narrow confidence interval)
1c	When all patients died before the measure was introduced, but some patients now survive on it.
2a	Systematic review of cohort studies (homogeneity)
2b	Individual cohort studies (including low-quality randomized controlled trials; e g., follow-up rate < 80%)
2c	A study of the outcome; an ecological study
3a	Systematic review of case–control studies (homogeneity)
3b	Individual case-control study
4	Case series (and poor-quality cohort studies and case–control studies)
5	Lack of clear and strictly evaluated expert advice, or derived from physiology, laboratory research, or “first principles”

**Level of recommended**	**Definition**

A	Evidence of consistent level 1
B	Consistent level 2 or 3 evidence, or extrapolation based on level 1 evidence. (“extrapolation” means that data are applied to situations with potentially clinically important differences rather than the original research)
C	Level 4 evidence, or extrapolation based on level 2 or 3 evidence
D	Level 5 evidence, either inconsistent or inadequate research (any level)

OCEBM, Oxford University Center for Evidence-Based Medicine.

### Definition

Trigeminal neuralgia is a typical neuropathic pain. ICD-11(International Classification of Diseases11) defines trigeminal neuralgia as “a unilateral disease characterized by transient electrical shock-like pain, with sudden onset and resolution, and the location of the pain is limited to one or more innervation areas of the trigeminal nerve” (Jones et al., 2019; Scholz et al., 2019a).

### Epidemiology

Approximately 12–29/100,000 people suffer from trigeminal neuralgia every year (Tai and Nayar, 2019). The incidence among women (5.9/100,000) is slightly higher than that among men (3.4/100,000) (Obermann and Katsarava, 2009). Trigeminal neuralgia can appear for the first time at any age, but more than 90% of cases have an onset age after 40, and the peak age is between 50 and 60 years old (MacDonald et al., 2000). The right side of the face is more affected than the left side (Cruccu et al., 2020).

### Pathogenesis

Trigeminal neuralgia is mainly divided into primary and secondary neuralgia. Primary neuralgia is divided into classic and idiopathic trigeminal neuralgia. The mechanism of primary trigeminal neuralgia is unknown. The main hypothesis of classic trigeminal neuralgia is that the trigeminal nerve root is compressed by abnormal blood vessels, which causes morphological changes in the trigeminal nerve. This may be related to ectopic impulses caused by demyelination and remyelination of the trigeminal nerve after compression (Cruccu et al., 2016; Tai and Nayar, 2019). Secondary trigeminal neuralgia is caused by identifiable diseases, such as trauma, tumors, viruses, and infections.

## Diagnosis and treatment

### Clinical manifestations

Trigeminal neuralgia is characterized mostly by a chronic course of illness that gradually worsens, and there may be a period of symptomatic relief. The pain is located in the distribution area of one or more branches of the unilateral trigeminal nerve. The nature of the pain is mostly that of an electric shock or knife cut that is. characterized by a paroxysmal onset and sudden cessation. The duration is usually several seconds to several minutes, and there is no pain in the intermittent period. Chewing, brushing, and touching can trigger pain. On physical examination, the “trigger point” can be elicited in the area of pain; trigger points are mostly located beside the nose, upper and lower lips, and gums.

### Imaging examination

Head CT or MRI can determine whether there is secondary trigeminal neuralgia caused by intracranial lesions, such as cerebellopontine angle-occupying lesions (1b evidence level, A level recommendation) (Chun-Cheng et al., 2009). Three-dimensional time-of-flight magnetic resonance angiography (3D-TOFMRA) can help clarify the anatomical relationship between the trigeminal nerve root and surrounding blood vessels(2b evidence level, B level recommendation) (Cha et al., 2011). Although it cannot be used as a diagnostic basis for primary trigeminal neuralgia, it can help guide the formulation of treatment plans (Bendtsen et al., 2020).

### Diagnosis and differential diagnosis

The diagnosis of primary trigeminal neuralgia is mainly based on clinical manifestations. You can refer to the following diagnostic criteria for trigeminal neuralgia in ICHD-3 of the International Headache Association:

(1) Recurrent unilateral pain occurs in one or more branches of the trigeminal nerve, does not involve the trigeminal nerve outside the distribution area, and fully meets the criteria of (2) and (3).

(2) Pain meets all of the following characteristics:

➀ It lasts from a few tenths of a second to 2 min;

➁ Severe pain;

➂ The nature of the pain is lightning, electric shock, acupuncture or sharp pain.

(3) Harmless stimuli on the affected side can induce pain.

(4) Other ICHD-3 diagnoses cannot provide a better explanation for the pain.

The differential diagnosis includes glossopharyngeal neuralgia, sphenopalatine neuralgia, cluster headache, atypical facial pain, and temporomandibular joint pain.

### Treatment

If the effect is not satisfactory or an adverse drug reaction cannot be tolerated, the choices for the treatment of trigeminal neuralgia include minimally invasive intervention, surgery, and gamma knife treatment.

(1) Drug treatment: Carbamazepine is the first-line choice, followed by oxcarbazepine. To avoid the risk of serious adverse reactions, it is recommended that drug genetic tests be performed before taking the drug when possible. If the patient cannot tolerate the side effects of the above drugs, other drugs can be used, such as pregabalin, gabapentin, phenytoin and sodium valproate. If the pain is persistent, a combination of medications can be used.

(2) Minimally invasive interventional treatment: Therapies include image-guided trigeminal nerve branch block or RF therapy, trigeminal nerve semilunar ganglion RF, chemical damage, or PMC. A minimally invasive interventional treatment technique is easy to perform, causes less trauma, is less costly and reliable. The main complications are hypoesthesia, keratitis, and masticatory muscle disorders.

(3) Surgical treatment: Surgeries include trigeminal nerve MVD, selective trigeminal nerve sensory roototomy, and peripheral neurotomy. MVD may be a non-destructive but highly traumatic surgical method for trigeminal neuralgia, with high immediate satisfaction of patients, a low incidence of postoperative complications such as facial numbness and weakness in mastication, and a complete pain relief rate of more than 90% after surgery, and 71% after 10 years (Sarsam et al., 2010). However, patients need to bear the risk of craniotomy, and high treatment costs, and frail elderly patients with more comorbidities have a higher risk. Surgical risks mainly include loss of facial sensation, hearing loss, cerebrospinal fluid leakage, and cerebellar hematoma.

(4) Gamma knife treatment: Gamma knife treatment for trigeminal neuralgia is widely used in the clinic, but there are few randomized controlled trials in clinical studies. Gamma knife treatment often does not relieve pain immediately and can take 1–2 months. At 3, 5, 7, and 10 years after treatment, the probabilities of remaining pain-free without the use of drugs were 77.9%, 73.8%, 68%, and 51.5%, respectively; the incidence of facial sensory disturbances was 20.8% (Régis et al., 2016).

(5) Other treatments include acupuncture, traditional Chinese medicine, and botulinum toxin injection.

## Minimally invasive interventional therapy

### Radiofrequency treatment

The common method of percutaneous RF involves puncture through the foramen ovale to reach the semilunar ganglion and temperature control to thermally coagulate the trigeminal nerve (Rabb and Cheema, 2015).

Sweet (1974) first reported that RF was used to treat primary trigeminal neuralgia. After years of improvement and development, this technique has many advantages, such as good curative effects, less trauma, low risk, and repeatable treatment (2a evidence level, B level recommendation) (Yan et al., 2021). RF, is indicated for patients with contraindications to surgery, especially elderly patients with underlying diseases. According to reports, during the 20-year follow-up, 41% of patients underwent a single operation, 100% of multiple operations resulted in pain relief, and there were no deaths (Kanpolat et al., 1999). Mathews (Mathews and Scrivani, 2000) reported the results of a study of 258 cases with an average follow-up of 38 months. Among them, the rate of excellent pain relief in the early follow-up was 87%. At the end of the long-term follow-up, 83% of patients had good pain relief.

The most commonly used puncture approach of RF for trigeminal neuralgia is the Hartel approach, which refers to the precise positioning of the foramen ovale and puncture path through X-ray or CT and selective puncturing into the corresponding trigeminal ganglion. Combined with electrophysiological stimulation for precise positioning tests, selective thermocoagulation damages the corresponding area of the trigeminal nerve semilunar ganglion to achieve analgesia (Fang et al., 2014). In addition to the commonly used semilunar radiofrequency, radiofrequency for the peripheral branch of the extracranial trigeminal nerve can also be selected to relieve the pain. The puncture sites include the supraorbital foramen, infraorbital foramen, mental foramen, circular foramen, and foramen ovale. In particular, RF for precise positioning of the circular hole under image guidance provides a good treatment option for patients with pain involving only the second branch of the trigeminal nerve (V2). When multiple branches are painful, multitarget radiofrequency treatment can be used (Wan et al., 2018).

(1) Indications
  ➀ Patients with poor drug-protection treatment for primary trigeminal neuralgia or those who are unable to tolerate adverse drug reactions;
  ➁ Patients with severe systemic diseases who cannot tolerate surgery or who refuse surgery;
  ➂ Patients with secondary trigeminal neuralgia who still have pain after treatment of the primary disease or who refuse treatment of the primary disease;
  ➃ Patients who relapse after various operations (Huang et al., 2019; Abdel-Rahman et al., 2020).

(2) Contraindications (Emril and Ho, 2010)
  ➀ Patients with infection at the puncture site;
  ➁ Patients with coagulation dysfunction;
  ➂ Patients with severely unstable heart and cerebrovascular diseases;
  ➃ Patients with severe mental illness.

(3) Operating routine

➀ Preoperative preparation

Tests such as routine blood tests, coagulation studies, biochemical examinations, and electrocardiograms should be performed to assess the patient’s body condition, along with head CT or MRI examination. The patients and their families should be fully informed of the treatment methods, expected results and possible complications before surgery.

➁ Methods of anesthesia

Sedative and analgesic drugs are recommended during local anesthesia to reduce the pain of puncture; thus, the patient can obtain analgesia and maintain sufficient cognition to achieve sensory positioning during puncture. After the positioning is successful, intravenous general anesthesia can be implemented.

➂ Operative process

The patient is placed in a supine position, with a thin pillow under the shoulders, after which the head is tilted posteriorly. Routinely sterilized drapes are used. According to the Hartel approach, the puncture needle point is determined, usually at the intersection of the perpendicular line of the outer edge of the affected orbit and the interlabial line region. The foramen ovale is punctured under image guidance (C-arm, DSA or CT). In the axial view, the first branch of the trigeminal nerve semilunar ganglion radiofrequency target is located above the medial edge of the foramen ovale, the second branch is located at the medial edge of the foramen ovale, and the third branch is located in the center of the foramen ovale. In the lateral view, the second branch of the semilunar ganglion of the trigeminal nerve is approximately, located at the slope line, the first branch is located deeper, and the third branch is loacted shallow relative to the slope line. After the puncture is in place, the RF electrode is connected, and the impedance at 300∼500 Ω is observed. A sensory test with 0.1∼0.3 V high-frequency current stimulation is performed, and the corresponding nerve innervation area will experience numbness and pain symptoms. When the movement test begins at 0.1∼0.3 V, the third innervation area will appear on the masseter. For convulsions, the needle tip position can be adjusted appropriately according to the patient’s response during the stimulation process to make the stimulation area consistent with the branch area to be treated. After the electrophysiological test is completed, radiofrequency thermocoagulation is performed. The recommended temperature for the treatment of the second and third branches is 60∼75°C. The first branch should be treated with caution. The time of treatment is based on the decrease in sensation of the target nerve distribution area on the affected side, usually 2∼3 min. The higher the temperature is, the longer the time, the greater the extent of nerve destruction, and the lower the recurrence rate. However, this treatment can induce a higher probability of adverse complications, such as keratitis or ulcer perforation, excessive numbness of the corresponding innervation area of the trigeminal nerve, and masseter muscle weakness after the operation (Xie et al., 2020). Therefore, during the treatment process, the patient’s treatment benefits and risks should be weighed, and as low a temperature as possible should be selected while still being effective. In addition, pulsed radiofrequency therapy is available (Elawamy et al., 2017; Telischak et al., 2018; Abdel-Rahman et al., 2020; Xie et al., 2020).

(4) Postoperative test and effect

Generally, immediate pain relief can be obtained, and the original pain area has decreased sensation after the operation.

(5) Matters needing attention
  ➀ Special attention should be given to changes in the operation center rate and blood pressure (Meng et al., 2008; Schaller et al., 2008).
  ➁ Accurate display of the foramen ovale and precise puncture guarantee the success of interventional therapy (Guo et al., 2016; Weßling and Duda, 2019).
  ➂ The RF temperature should be strictly controlled to avoid excessive temperature-induced damage to the target nerve and adjacent branches, which can cause postoperative facial numbness, masseter muscle weakness and other complications (Yao et al., 2016). If the corneal reflex is delayed after surgery, medical care should be implemented to avoid keratitis, ulcers, and perforations (Tang et al., 2015).

### Percutaneous microcompression

PMC of the meniscus ganglion refers to the treatment of trigeminal neuralgia by placing a balloon into Meckel’s cavity and injecting a contrast agent into the balloon to mechanically compress the meniscus. Mullan and Lichtor (1983) first used this technique to treat trigeminal neuralgia in 1978 and published his research paper in 1983 (Mullan and Lichtor, 1983). At present, the treatment mechanism of PMC is not clear. In recent years, because this technology is safe and effective, it has been widely used in the treatment of primary trigeminal neuralgia (1a evidence level, A level recommendation) (Wu et al., 2022). Grewal et al. (2018) conducted a median follow-up of 31.1 months for 222 patients with refractory trigeminal neuralgia who were treated using PMC, and all patients had remission within 12 months after PMC. It has been reported that the 5-year effective rate after surgery is 80%, and the 10-year effective rate is 70% (Lichtor and Mullan, 1990). Fan et al. conducted a 12 months follow-up of study of 121 patients with recurrent trigeminal neuralgia treated using PMC. It has been reported that 101 (83.5%) patients remained pain-free, while 5 patients (4.1%) experienced recurrence. There were significant improvements in anxiety, depression, and sleep status postoperatively compared with the preoperatively status (Fan et al., 2021).

(1) Indications

In recent years, clinical practices have shown that PMC has a wide range of indications, especially for patients with pain involving the first branch of the trigeminal nerve. Because PMC selectively acts on large and medium myelinated nerve fibers, it can reduce the impairment of nerves (small, myelinated fibers) innervating the corneal reflex (Cheng et al., 1982).

The indications include the following

➀ Patients with primary trigeminal neuralgia involving single or multiple branches, especially within the first branch;

➁ Patients who have undergone strict and regular drug treatment with poor outcomes and those cannot tolerate adverse reactions;

➂ Imaging data suggesting that microvascular decompression will be relatively difficult or risky in patients who do not undergo craniotomy;

➃ Patients who relapse after other surgery (Fan et al., 2021);

➄ Patients with secondary trigeminal neuralgia who still have pain after treatment of the primary disease or who refuse treatment of the primary disease (Liu, 2018).

(2)Contraindications

➀ Patients with severe systemic diseases who cannot tolerate surgery;

➁ Patients with uncorrected coagulation dysfunction;

➂ Severe systemic infection or infection at the puncture site.

(3) Operating routine

➀ Preoperative preparation

Before surgery, routine blood tests, routine blood coagulation tests, biochemical examinations, electrocardiograms, head CT or MRI examinations, etc., should be performed to assess the whole body and local nerve condition. Patients provided full informed consent which covers the treatment methods, expected results and possible complications before surgery.

➁ Anesthesia method

General anesthesia: The advantage of general anesthesia is that the patient is unconscious and out of pain during the operation, but the disadvantage is that some patients with systemic diseases cannot tolerate it, and the incidence of trigeminal nerve-cardiac reflex is relatively high (Tibano et al., 2010; Agarwal et al., 2015).

General anesthesia-assisted trigeminal ganglion block: the advantages of general anesthesia can be retained, and the incidence of the trigeminal nerve-cardiac reflex is lower than that of general anesthesia alone (Tibano et al., 2010).

Trigeminal ganglion block anesthesia: The puncture point and puncture direction are the same as those under general anesthesia. The local anesthetic needle is used for local anesthesia at the puncture point and needle channel. Under the guidance of imaging, the puncture needle enters through the foramen ovale to reach the ganglion, and 0.3∼0.5 ml of the local anesthetics is injected for the ganglion block. This method is suitable for patients who cannot tolerate general anesthesia, and the incidence of the trigeminal nerve-cardiac reflex is low (Ren et al., 2020). The disadvantage is that when some patients are awake and experience anxiety, but they can administered.

➂ Operative process

After the image guide reaches the ideal position, non-ionic contrast agent is injected, with a dose of usually no more than 1 ml, and the pressure is adjusted to the balloon such that resistance is experienced. The position is adjusted until the balloon shows a “pear shape.” The balloon compression time is usually 60–180 s (Tibano et al., 2010; Bergenheim et al., 2013; de Cordoba et al., 2015; Grewal et al., 2018), and it can be extended appropriately for elderly or relapsed patients. After the compression is completed, the contrast medium in the balloon is withdrawn, and the balloon catheter and puncture needle are removed. The puncture point is compressed for 5 min to stop the bleeding and then is covered with sterile gauze.

(4) Postoperative test and effect

The ideal balloon filling shape should be a “pear shape.” The affected side will have decreased sensation after surgery, and pain relief can generally be achieved. If the balloon shape is not ideal, the puncture position and direction should be adjusted (Asplund et al., 2010).

(5) Matters needing attention

➀ Preoperative MRI thin-slice scans can be used to estimate the volume of Meckel’s cavity. The volume of the contrast medium injected during the operation should be greater than the estimated volume of Meckel’s cavity, but it must be subject to obvious resistance when the contrast medium is injected.

➁ During balloon compression, a trigeminal nerve-cardiac reflex may occur. It is necessary to closely monitor changes in heart rate and blood pressure and address changes in a timely manner.

➂ During the operation, advancing the puncture needle too deeply and allowing it to deviate to the inside should be avoided (Spaziante et al., 1988). Damage to the internal carotid artery, cavernous sinus or other cranial nerves should be avoided.

➃ The incidence of postoperative perioral herpes is 14.7%, and preventive antiviral therapy may reduce its incidence (Berra et al., 2019).

➄ After PMC, the sensations in the oral cavity on the operative side are decreased. Overheated or sharp foods should be avoided to prevent damage to the oral mucosa.

### Other interventional treatments

In addition to the abovementioned mechanical compression or physical damage methods, minimally invasive treatment of primary trigeminal neuralgia employs chemical injections into the semilunar ganglia, trigeminal nerve pool or peripheral branches for chemical damage. Chemical drugs include absolute ethanol, glycerin, botulinum toxin A and doxorubicin. Among them, the effect of glycerol on the semilunar ganglion and trigeminal nerve pool is supported by domestic and foreign literature (2b evidence level, B level recommendation) (Chen et al., 2010; Asplund et al., 2016; Staudt et al., 2020). Anhydrous alcohol can be used for the semilunar ganglion and peripheral division (2b evidence level, B level recommendation) (Han and Kim, 2010; Han et al., 2017), and botulinum toxin A (1a evidence level, A level recommendation) (Morra et al., 2016; Steinberg, 2018; Yang et al., 2018) and doxorubicin are only used in the peripheral branches (Wang et al., 2011; Zheng et al., 2018); these drugs have a damaging effect on the surrounding tissues, and their range of action is not easy to control, which may cause serious injuries. At present, with the popularization and application of technologies such as radio frequency and microballoon compression, the use of chemical damage has become increasingly less common (Udupi et al., 2012; Asplund et al., 2016; Noorani et al., 2016, 2019), and it is only used at the grassroots level or on specific occasions. It is recommended that chemical damage in the peripheral branches of the trigeminal nerve be used as a supplementary treatment for other therapies. It should be used with caution in the intracranial semilunar ganglion. When used in peripheral branches, attention should be given to signs of local tissue damage, such as swelling.

In summary, among these minimally invasive interventional treatments for trigeminal neuralgia, RF and PMC are the most widely performed and effective treatments. The comparison of the characteristics of minimally invasive interventional treatment for trigeminal neuralgia is shown in Table 2. Precise imaging positioning and guidance are the key to ensuring the efficacy and safety of the procedures. X-ray has the advantages of being convenient, fast and involving low radiation, and it is easy to determine the shape of the balloon in the lateral view. 3D CT involves the use of a large amount of radiation, but it can help to analyze the size and shape of the foramen ovale and simulate the puncture path, and it is easier to observe the path of the RF needle and puncture needle, thus, it is suitable for beginners. Many clinicians have accumulated much experience in puncture procedures, but there are still many controversies about the key criteria of radiofrequency temperature, time, microballoon volume, compression time, and related factors. Further basic and clinical studies are needed to provide more accurate evidence to guide clinical applications.

**TABLE 2:** Comparison of minimally invasive interventional treatments of characteristics for trigeminal neuralgia.

Characteristics	Radiofrequency treatment	Percutaneous microcompression	Chemical damage
Anesthesia	Local anesthesia	General anesthesia/Local anesthesia	Local anesthesia
Operation difficulty	Low	Low	Low
Long-term curative effect	Medium	High	Medium
Recurrence rate	Low	Low	Medium
Degree of safety	High	High	Low
Complications	Medium	Low	Medium
Expenses	Low	Medium	Low

## Author contributions

All authors made significant contributions to the content of the article, drafted or critically revised the article, and ultimately approved the version to be published.

## Abbreviations

RF: radiofrequency thermocoagulation
PMC: Percutaneous microcompression

## References

[B1] Abdel-Rahman K. A. Elawamy A. M. Mostafa M. F. Hasan W. S. Herdan R. Osman N. M. (2020). Combined pulsed and thermal radiofrequency versus thermal radiofrequency alone in the treatment of recurrent trigeminal neuralgia after microvascular decompression: a double blinded comparative study. *Eur. J. Pain (United Kingdom)* 24 338–345. 10.1002/ejp.1489 31571339

[B2] Agarwal A. Dhama V. Manik Y. K. Upadhyaya M. K. Singh C. S. Rastogi V. (2015). Percutaneous balloon compression of gasserian ganglion for the treatment of trigeminal neuralgia: an experience from India. *Middle East J. Anesthesiol.* 23 105–110. 26121902

[B3] Asplund P. Blomstedt P. Tommy Bergenheim A. (2016). Percutaneous balloon compression vs percutaneous retrogasserian glycerol rhizotomy for the primary treatment of trigeminal neuralgia. *Neurosurgery* 78 421–428. 10.1227/NEU.0000000000001059 26465639 PMC4747977

[B4] Asplund P. Linderoth B. Bergenheim A. T. (2010). The predictive power of balloon shape and change of sensory functions on outcome of percutaneous balloon compression for trigeminal neuralgia: clinical article. *J. Neurosurg.* 113 498–507. 10.3171/2010.2.JNS091466 20345223

[B5] Bendtsen L. Zakrzewska J. M. Heinskou T. B. Hodaie M. Leal P. R. L. Nurmikko T. (2020). Advances in diagnosis, classification, pathophysiology, and management of trigeminal neuralgia. *Lancet Neurol.* 19 478–496. 10.1016/S1474-4422(20)30233-7 32822636

[B6] Bergenheim A. T. Asplund P. Linderoth B. (2013). Percutaneous retrogasserian balloon compression for trigeminal neuralgia: review of critical technical details and outcomes. *World Neurosurg.* 79 359–368. 10.1016/j.wneu.2012.03.014 22480980

[B7] Berra L. V. Armocida D. Pesce A. di Rita A. Santoro A. (2019). Herpes simplex reactivation after surgical treatment of trigeminal neuralgia: a retrospective cohort study. *World Neurosurg.* 127 e16–e21. 10.1016/j.wneu.2019.01.226 30771541

[B8] Cha J. Kim S. T. Kim H. J. Choi J. W. Kim H. J. Jeon P. (2011). Trigeminal neuralgia: assessment with T2 VISTA and FLAIR VISTA fusion imaging. *Eur. Radiol.* 21 2633–2639. 10.1007/s00330-011-2216-1 21822786

[B9] Chen L. Z. Xu M. H. Zou Y. W. (2010). Treatment of trigeminal neuralgia with percutaneous glycerol injection into Meckel’s cavity: experience in 4012 patients. *Cell Biochem. Biophys.* 58 85–89. 10.1007/s12013-010-9094-z 20717747

[B10] Cheng J. S. Lim D. A. Chang E. F. Barbaro N. M. (1982). A review of percutaneous treatments for trigeminal neuralgia. *Neurosurgery* 10 25–33. 10.1227/NEU.00000000000001687 24509496

[B11] Chun-Cheng Q. Qing-Shi Z. Ji-Qing Z. Zhi-Gang W. (2009). A single-blinded pilot study assessing neurovascular contact by using high-resolution MR imaging in patients with trigeminal neuralgia. *Eur. J. Radiol.* 69 459–463. 10.1016/j.ejrad.2007.10.010 18035511

[B12] Cruccu G. di Stefano G. Truini A. (2020). Trigeminal neuralgia. *N. Engl. J. Med.* 383 754–762. 10.1056/NEJMra1914484 32813951

[B13] Cruccu G. Finnerup N. B. Jensen T. S. Scholz J. Sindou M. Svensson P. (2016). Trigeminal neuralgia: new classification and diagnostic grading for practice and research. *Neurology* 87 220–228. 10.1212/WNL.0000000000002840 27306631 PMC4940067

[B14] de Cordoba J. L. Garcia Bach M. Isach N. Piles S. (2015). Percutaneous balloon compression for trigeminal neuralgia: imaging and technical aspects. *Reg. Anesth. Pain Med.* 40 616–622. 10.1097/AAP.0000000000000292 26236998

[B15] Elawamy A. Abdalla E. E. Shehata G. A. (2017). Effects of pulsed versus conventional versus combined radiofrequency for the treatment of trigeminal neuralgia: a prospective study. *Pain Phys.* 20 E873–E881. 28934792

[B16] Emril D. R. Ho K. Y. (2010). Treatment of trigeminal neuralgia: role of radiofrequency ablation. *J. Pain Res.* 3 249–254. 10.2147/JPR.S14455 21311718 PMC3033033

[B17] Fang L. Ying S. Tao W. Lan M. Xiaotong Y. Nan J. (2014). 3D CT-guided pulsed radiofrequency treatment for trigeminal neuralgia. *Pain Pract.* 14 16–21. 10.1111/papr.12041 23433058

[B18] Fan X. Xu F. Ren H. Lu Z. Bu H. Ma L. (2021). The analysis of percutaneous balloon compression on efficacy and negative emotion in the treatment of recurrent trigeminal neuralgia after surgical procedures. *Pain Phys.* 24 e1255–e1262. 34793652

[B19] Grewal S. S. Kerezoudis P. Garcia O. Quinones-Hinojosa A. Reimer R. Wharen R. E. (2018). Results of percutaneous balloon compression in trigeminal pain syndromes. *World Neurosurg.* 114 e892–e899. 10.1016/j.wneu.2018.03.111 29581020

[B20] Guo Z. Wu B. Du C. Cheng M. Tian Y. (2016). Stereotactic approach combined with 3D CT reconstruction for difficult-to-access foramen ovale on radiofrequency thermocoagulation of the gasserian ganglion for trigeminal neuralgia. *Pain Med. (United States)* 17 1704–1716. 10.1093/pm/pnv108 26874883

[B21] Han K. R. Chae Y. J. Lee J. D. Kim C. (2017). Trigeminal nerve block with alcohol for medically intractable classic trigeminal neuralgia: long-term clinical effectiveness on pain. *Int. J. Med. Sci.* 14 29–36. 10.7150/ijms.16964 28138306 PMC5278656

[B22] Han K. R. Kim C. (2010). The long-term outcome of mandibular nerve block with alcohol for the treatment of trigeminal neuralgia. *Anesth. Anal.* 111 550–553. 10.1213/ANE.0b013e3181e4204c 20584871

[B23] Huang B. Xie K. Chen Y. Wu J. Yao M. (2019). Bipolar radiofrequency ablation of mandibular branch for refractory V3 trigeminal neuralgia. *J. Pain Res.* 12 1465–1474. 10.2147/JPR.S197967 31190956 PMC6514122

[B24] Jones M. R. Urits I. Ehrhardt K. P. Cefalu J. N. Kendrick J. B. Park D. J. (2019). A comprehensive review of trigeminal neuralgia. *Curr. Pain Head. Rep.* 23:74. 10.1007/s11916-019-0810-0 31388843

[B25] Kanpolat Y. Savas A. Bekar A. Berk C. (1999). Percutaneous controlled radiofrequency trigeminal rhizotomy for the treatment of idiopathic trigeminal neuralgia: a 25-year experience with 1,600 patients. *Neurosurgery* 45 532–534. 10.1097/00006123-199909000-00209 11270542

[B26] Lichtor T. Mullan J. F. (1990). A 10-year follow-up review of percutaneous microcompression of the trigeminal ganglion. *J. Neurosurg.* 72 49–54. 10.3171/jns.1990.72.1.0049 2294184

[B27] Liu Q. (2018). Interpretation of “Chinese expert consensus on diagnosis and treatment of trigeminalneuralgia. *Chin. J. Contemp. Neurol. Neurosurg.* 18 643–646. 10.3969/j.issn.1672-6731.2018.09.003

[B28] MacDonald B. K. Cockerell O. C. Sander J. W. A. S. Shorvon S. D. (2000). The incidence and lifetime prevalence of neurological disorders in a prospective community-based study in the UK. *Brain* 123 665–676. 10.1093/brain/123.4.665 10733998

[B29] Mathews E. S. Scrivani S. J. (2000). Percutaneous stereotactic radiofrequency thermal rhizotomy for the treatment of trigeminal neuralgia. *Mt. Sinai J. Med.* 67 288–299. 11021779

[B30] Meng Q. Zhang W. Yang Y. Zhou M. Li X. (2008). Cardiovascular responses during percutaneous radiofrequency thermocoagulation therapy in primary trigeminal neuralgia. *J. Neurosurg. Anesthesiol.* 20:270. 10.1097/ANA.0b013e3181628305 18362775

[B31] Morra M. E. Elgebaly A. Elmaraezy A. Khalil A. M. Altibi A. M. A. Vu T. L. H. (2016). Therapeutic efficacy and safety of botulinum toxin a therapy in trigeminal neuralgia: a systematic review and meta-analysis of randomized controlled trials. *J. Head. Pain* 17:63. 10.1186/s10194-016-0651-8 27377706 PMC4932020

[B32] Mullan S. Lichtor T. (1983). Percutaneous microcompression of the trigeminal ganglion for trigeminal neuralgia. *J. Neurosurg.* 59 745–748. 10.3171/jns.1983.59.6.1007 6631493

[B33] Noorani I. Lodge A. Vajramani G. Sparrow O. (2016). Comparing percutaneous treatments of trigeminal neuralgia: 19 years of experience in a single centre. *Stereotact. Funct. Neurosurg.* 94 75–85. 10.1159/000445077 27071078

[B34] Noorani I. Lodge A. Vajramani G. Sparrow O. (2019). The effectiveness of percutaneous balloon compression, thermocoagulation, and glycerol rhizolysis for trigeminal neuralgia in multiple sclerosis. *Clin. Neurosurg.* 85 E684–E692. 10.1093/neuros/nyz103 30957177

[B35] Obermann M. Katsarava Z. (2009). Update on trigeminal neuralgia. *Exp. Rev. Neurother.* 9 323–329. 10.1586/14737175.9.3.323 19271941

[B36] Rabb C. Cheema A. (2015). “Trigeminal neuralgia: viewpoint—surgery,” in *Principles and Practice of Stereotactic Radiosurgery*, eds Chin L. Regine W. (New York, NY: Springer), 659–664. 10.1007/978-1-4614-8363-2_53

[B37] Régis J. Tuleasca C. Resseguier N. Carron R. Donnet A. Yomo S. (2016). The very long-term outcome of radiosurgery for classical trigeminal neuralgia. *Stereotact. Funct. Neurosurg.* 94 24–32. 10.1159/000443529 26882097

[B38] Ren Y. Han W. du Y. Cong H. Liu G. (2020). Efficacy and safety of CT-guided percutaneous microballoon compression in the treatment of patients with primary trigeminal neuralgia under conscious trigeminal ganglion local block. *Chine. J. Painol.* 1 30–35. 10.3760/cma.j.issn.2096-8019.2020.01.009 30704229

[B39] Sarsam Z. Garcia-Fiana M. Nurmikko T. J. Varma T. R. K. Eldridge P. (2010). The long-term outcome of microvascular decompression for trigeminal neuralgia. *Br. J. Neurosurg.* 24 1077–1083. 10.3109/02688690903370289 20158348

[B40] Schaller B. Sandu N. Fili A. Buchfelder M. (2008). Cardiovascular responses during percutaneous radiofrequency thermocoagulation therapy in primary trigeminal neuralgia: an explanation of the trigeminocardiac reflex? *J. Neurosurg. Anesthesiol.* 20:270. 10.1097/ANA.0b013e3181817b50 18812892

[B41] Scholz J. Finnerup N. B. Attal N. Aziz Q. Baron R. Bennett M. I. (2019a). The IASP classification of chronic pain for ICD-11: chronic neuropathic pain. *Pain* 160 53–59. 10.1097/j.pain.0000000000001365 30586071 PMC6310153

[B42] Spaziante R. Cappabianca P. Peca C. de Divitiis E. (1988). Subarachnoid hemorrhage and “normal pressure hydrocephalus”: fatal complication of percutaneous microcompression of the gasserian ganglion: case report. *Neurosurgery* 22 148–151. 10.1227/00006123-198801000-00028 3344077

[B43] Staudt M. D. Joswig H. Pickett G. E. MacDougall K. W. Parrent A. G. (2020). Percutaneous glycerol rhizotomy for trigeminal neuralgia in patients with multiple sclerosis: a long-term retrospective cohort study. *J. Neurosurg.* 132 1405–1413. 10.3171/2019.1.JNS183093 30978686

[B44] Steinberg D. I. (2018). Review: in trigeminal neuralgia, carbamazepine, botulinum toxin type A, or lidocaine improve response rate vs placebo. *Ann. Intern. Med.* 169:43. 10.7326/ACPJC-2018-169-8-043 30326087

[B45] Tai A. X. Nayar V. V. (2019). Update on trigeminal neuralgia. *Curr. Treat. Opt. Neurol.* 21:42. 10.1007/s11940-019-0583-0 31367794

[B46] Telischak N. A. Heit J. J. Campos L. W. Choudhri O. A. Do H. M. Qian X. (2018). Fluoroscopic C-arm and CT-guided selective radiofrequency ablation for trigeminal and glossopharyngeal facial pain syndromes. *Pain Med. (United States)* 19 130–141. 10.1093/pm/pnx088 28472393

[B47] Tibano A. T. de Siqueira S. R. D. T. da Nóbrega J. C. M. Teixeira M. J. (2010). Cardiovascular response during trigeminal ganglion compression for trigeminal neuralgia according to the use of local anesthetics. *Acta Neurochir.* 152 1347–1351. 10.1007/s00701-010-0664-z 20473771

[B48] Udupi B. P. Chouhan R. S. Dash H. H. Bithal P. K. Prabhakar H. (2012). Comparative evaluation of percutaneous retrogasserian glycerol rhizolysis and radiofrequency thermocoagulation techniques in the management of trigeminal neuralgia. *Neurosurgery* 70 407–412. 10.1227/neu.0b013e318233a85f 21866065

[B49] Wan Q. Zhang D. Cao X. Zhang Y. Zhu M. Zuo W. (2018). CT-guided selective percutaneous radiofrequency thermocoagulation via the foramen rotundum for isolated maxillary nerve idiopathic trigeminal neuralgia. *J. Neurosurg.* 128 211–214. 10.3171/2016.9.JNS152520 28298043

[B50] Wang H. Zheng B. Shi K. Liu J. Ma W. Liu Q. (2011). The effect of adriamycin interventional therapy for patients with trigeminal neuralgia of ophthalmic branch guided by X ray and nerve stimulator. *Chine. J. Pain Med.* 5 286–290. 10.3969/j.issn.1006-9852.2011.05.009

[B51] Weßling H. Duda S. (2019). ioCT-guided percutaneous radiofrequency ablation for trigeminal neuralgia: how I do it. *Acta Neurochir.* 161 935–938. 10.1007/s00701-019-03859-8 30911830

[B52] Wu J. Xiao Y. Chen B. Zhang R. Dai M. Zhang Y. (2022). Efficacy and safety of microvascular decompression versus percutaneous balloon compression in the treatment of trigeminal neuralgia: a systematic review and meta-analysis. *Ann. Palliat. Med.* 11 1391–1400. 10.21037/apm-21-3901 35523747

[B53] Xie K. Liu S. Huang B. Yao M. (2020). Effects of supraorbital foramen variations on the treatment efficacy of radiofrequency therapy for V1 trigeminal neuralgia: a retrospective study. *Pain Res. Manage.* 2020:8142489. 10.1155/2020/8142489 32184911 PMC7061117

[B54] Yan C. Zhang Q. Liu C. Yang J. Bian H. Zhu J. (2021). Efficacy and safety of radiofrequency in the treatment of trigeminal neuralgia: a systematic review and meta-analysis. *Acta Neurol. Belg.* 10.1007/s13760-021-01654-w 33988820

[B55] Yang F. Lin Q. Dong L. Gao X. Zhang S. (2018). Efficacy of 8 different drug treatments for patients with trigeminal neuralgia: a network meta-analysis. *Clin. J. Pain* 34 685–690. 10.1097/AJP.0000000000000577 29200017

[B56] Yao P. Hong T. Wang Z. B. Ma J. M. Zhu Y. Q. Li H. X. (2016). Treatment of bilateral idiopathic trigeminal neuralgia by radiofrequency thermocoagulation at different temperatures. *Medicine (United States)* 95:E4274. 10.1097/MD.0000000000004274 27442662 PMC5265779

[B57] Tang Y. Z. Wu B. S. Yang L. Q. Yue J. N. He L. L. Li N. (2015). The long-term effective rate of different branches of idiopathic trigeminal neuralgia after single radiofrequency thermocoagulation. *Medicine (United States)* 94:e1994. 10.1097/MD.0000000000001994 26559288 PMC4912282

[B58] Zheng B. Song L. Liu H. (2018). Gasserian ganglion injected with adriamycin successfully relieves intractable trigeminal nerve postherpetic neuralgia for an elderly patient: a case report. *Medicine (United States)* 97:e12388. 10.1097/MD.0000000000012388 30235706 PMC6160063

